# Supplementary material for: Diet changes due to urbanization in South Africa are linked to microbiome and metabolome signatures of Westernization and colorectal cancer
Source: Nat Commun. 2024 Apr 20;15:3379. doi: 10.1038/s41467-024-46265-0 (PMC11032404; doi:10.1038/s41467-024-46265-0)
Supplement: Supplementary file 1 — Supplementary Information [file 41467_2024_46265_MOESM1_ESM.pdf]

SUPPLEMENTARY METHODS

Study population

For the n=44 participants who enrolled into the study in 2020, the following inclusion criteria were applied: informed consent; age of 40-75 years; self-identify as AmaXhosa; BMI between 20-40 kg/m^2. Exclusion criteria were: previous GI surgery resulting in disturbed gut function due to loss of bowel or altered anatomy; any form of chronic GI disease resulting in disturbed gut function, diarrhea, or malabsorption; any form of acute GI disease disturbing GI function and needing current medication, e.g. gastroenteritis, peptic ulcer disease; history of any GI malignancy; present GI malignancy, previously known or detected at screening endoscopy; presence of any other form of cancer or malignancy; oral or i.v. antibiotic therapy within the last 6 weeks; diabetes (self-reported).

The Zithulele village setting has been previously described [1,2]: briefly, Zithulele lies in the Mqanduli magisterial area of the King Sabata Dalindyebo sub-district, part of the Oliver Tambo district, in the Eastern Cape province of South Africa. It is a semi-rural, hilly coastal village embedded in the poorest and most under-developed district in the country. Its basic infrastructure includes a hospital, that serves a population of about 150,000 in an area of nearly 1,000 km^2, a research center, schools, shopping center, as well as the roads and main electricity line connecting these places. Many households lack access to electricity, safe running water and sanitation, and depend on government grants and food produced through subsistence.

^{1}H NMR spectroscopy-based metabolic profiling of urine and fecal water

To prepare fecal water for ^{1}H NMR spectroscopic analysis, two volumes of HPLC-grade water (~1.2 ml) were added to freeze-thawed raw fecal samples (~600 mg) with a ratio of 2:1. The slurry was then vortexed at 2500 rpm for 5 mins and centrifuged at 18,000 *g* for 15mins. All the clear supernatant (fecal water) was transferred into a new tube and stored at -80℃ until NMR analysis. Urinary samples were defrosted and centrifuged at 12,000 *g* at 4°C for 10 mins, and 540 µL of supernatant was introduced into an NMR tube with an outer diameter of 5 mm and mixed with 60 µL of phosphate buffer containing 100% deuterium oxide (D_2O), 1.5 M of KH_2PO_4, 5.8 mM of 3-trimethyl-silyl-[2,2,3,3-$^2H_4$] propionic acid (TSP) and 2 mM of NaN_3 at pH 7.4 for standard 1 dimensional ^{1}H NMR NOESY analysis. Fecal water was defrosted at room temperature for 30 min. The samples were centrifuged at 18,000 *g* for 10 min at 4℃. Four hundred and fifty microliters of the clean supernatant were mixed with 90 µL of HPLC-grade water and 60 µL of the phosphate buffer in a new tube by vortexing for 5 s, and 580 µL were transferred to an NMR tube with an outer diameter of 5 mm for standard 1D ^{1}H NOESY analysis. ^{1}H NMR spectra of urinary and fecal water extract samples were acquired as described previously [3]. TSP and water components were removed before applying for alignment and probabilistic quotient normalisation [4,5]. Multivariate statistical analyses including principal component analysis (PCA) and orthogonal projections to latent structures discriminant analysis (OPLS-DA) were performed to investigate differences in the global fecal and urinary metabolite profiles.

Quantitation of Amino Acids by Ultra High-Performance Liquid Chromatography with Mass Spectrometry (UPLC-MS/MS)

Preparation of amino acids standard solutions

Alanine, arginine, aspartate, carnosine, citrulline, cystathionine, cysteine, glutamate, glutamine, glutathione, glycine, histidine, homocysteine, isoleucine, kynurenine, leucine, lysine, methionine, ornithine, phenylalanine, sarcosine, serine, taurine, threonine, tryptophan, tyrosine with 98-99% purity were purchased from Sigma-Aldrich (Steinheim, Germany). Isotope-labelled internal standards isoleucine-D10, leucine-D10, lysine-D4, methionine-D3 and threonine 15N-13C4 were purchased from Cambridge Isotopes Laboratories Inc. (Tewksbury, Massachusetts, USA). Labelled and non-labelled amino acid stock solutions were prepared at a concentration of 1 M, in acetonitrile (Optima LCMS grade, Fisher Scientific, Leicester, UK) at 10% v/v with 3 M perchloric acid (Sigma-Aldrich, Steinheim, Germany) to the final solution volume; then stored at -80°C in silanized glass vials. Serial dilutions in acetonitrile from 1 M stock solutions were prepared to produce 10 concentrations (100, 50, 25, 10, 5, 2.5, 1, 0.5, 0.25, 0.1 and 0 µM) to establish calibration curves. Labelled internal standards were gradually diluted from stock solutions at 1 M into a mixture solution to obtain final concentrations of 20 µM.

Extraction of amino acids from faecal samples

All samples were mixed by using a spatula prior to transferring 10-50 mg to a 2 mL polypropylene cryovial (Starlab, Milton Keynes, UK) containing approximately 100 mg of 0.5-1 mm diameter glass beads (BioSpec Products, Bartlesville, Oklahoma, USA). This initial wet weight (Wo) of each sample was recorded. Additionally, 100 µL of 100 mM 1,4 dithioerythritol (Sigma-Aldrich, Steinheim, Germany), 100 µL of 3 M perchloric acid (Sigma Aldrich, Steinheim, Germany) and 800 µL methanol (Optima LCMS grade, Fisher Scientific, Leicester, UK) were aliquoted into the cryovial containing the sample and mixed for few seconds. The vial was left at room temperature for 30 minutes to incubate, then it was placed in a bead beater (Precellys, Bertin Technologies, Montigny Le Bretonneux, France) for homogenisation applying three cycles at 6000 rpm for 30 seconds per cycle. Homogenized sample was centrifuged at 16,000 g for 15 minutes at 4°C (Eppendorf 5430R Centrifuge, Hamburg, Germany). The supernatant was transferred to a sterile 2 mL polypropylene cryovial (Starlab, Milton Keynes, UK) and stored at -80°C until derivatisation.

Derivatisation

Fecal extracts were diluted five-fold in acetonitrile before derivatisation following a modified methodology using 4-dimethyl phenacyl amino bromide (DmPABr) published before [1]. A 10 µL aliquot of diluted faecal extracts, 10 µL of internal standard solution mixture, 20 µL 0.75M triethanolamine (Sigma Aldrich, Hamburg, Germany) and 20 µL 80 mM DmPABr (Apollo Scientific, Cheshire, UK) were added to 1.5 mL microcentrifuge tubes (Eppendorf, Hamburg, Germany). The samples were vortexed for few seconds and incubated at 65°C for one hour (ThermoMixer C, Eppendorf, Hamburg, Germany). After incubation, 20 µL of 30mg/ml formic acid solution and 90 µL acetonitrile was added to the tubes which were then centrifuged at 16,000g for 5 minutes (Eppendorf 5430R Centrifuge, Hamburg, Germany). Supernatants were transferred to clear glass vials with a 250 µL insert (Agilent, Santa Clara, California, USA) and stored at 4°C until injection. Standard calibration curves were derivatised following the same procedure.

Ultra-High Performance Liquid Chromatography with Tandem Mass Spectrometry (UHPLC-MS/MS)

A reverse phase (RP) chromatographic separation was performed on a Waters Acquity Binary Solvent Management (BSM) UHPLC system (Milford, USA) using a Waters CSH C18 Column with dimensions 100 mm × 2.1 mm, 1.7 µm particle size, 130 Å pore size (Milford, USA). Mobile phase A (MPA) consisted of 0.1% formic acid (Supelco, Bellefonte, USA) solution in ultrapure water (Optima LCMS Grade, Fisher Scientific, Leicester, UK). Mobile phase B (MPB) consisted of 100% acetonitrile (Optima LCMS grade, Fisher Scientific, Leicester, UK). Total running time was 15 minutes. Chromatographic separation started with flow rate of 0.75 mL/min at 99.8 % MPA and 0.2% MPB; 3 min, 0.2% MPB; 5 min, 55% MPB; 8 min, 99% MPB; 13 min, 99% MPB; 13.5 min, 0.2% MPB; and 15 min, 0.2% MPB. Column temperature was kept constant at 60°C and the injection loop volume was 1 µL.

Amino acids were detected on a Waters Xevo TQ-S (Waters, Milford, USA) on negative ESI polarity. Intellistart Software (Waters, Milford, USA) produced reports for four most abundant ion pair transitions selected from MS scans in Q1 (the first set of quadrupoles) and fragmented ions obtained in Q3 (the set of quadrupoles after collision energy has been applied) as Q1>Q3 for each derivatized compound. In our developed method, data was obtained using segmented Multiple Reaction Monitoring (MRM). For each compound, one MRM transitions were acquired with a detection window of 30 seconds either side of the retention time. Dwell time was automatically adjusted to 100 milli seconds. Mass Spectrometry (MS) data was acquired using MassLynx V4.2 software (Waters, Milford, USA) and processed using TargetLynx XS software (Waters, Milford, USA). The MS capillary voltage was set at 2000 V; cone voltage, 60 V; cone temperature, 150°C; desolvation gas flow, 1000 L/h; and desolvation temperature, 500°C. Ion pair masses (Q1>Q3) and fragmentation patterns were verified by using ChemDraw Pro (Version 19.01.08, Perking Elmer Informatics Inc, Waltham, MA, USA).

Food metabolomic analysis by LC-MS/MS

Metabolite extraction for food samples

Each food sample was weighed out (~100 mg) into a 2 mL tube. Then 1 mL of 3:1 hexane:MeOH (v/v) solution was added into each tube and each sample was vortexed for 3 minutes. The samples were put

on a shaker for 10 minutes at 1000 rpm. The samples were then sonicated on an ice bath for 10 minutes using a sonicator. After sonication, 650 uL of 3:1 H_2O:MeOH (v/v) was added to each tube. All samples were then again vortexed for one minute. Then samples were then centrifuged for 10 minutes at 10,000x *g* at 4^0C. After centrifuging, samples were separated into two layers. The top layer (organic part) was transferred into a new 1.5 mL tube. The samples were completely dried using nitrogen purge for 20 minutes. The dried samples were then reconstituted by adding acetonitrile to the tubes. The reconstituted samples were stored at -20 0C freezer until LC-MS/MS analysis.

TMIC Prime Assay DI/LC-MS/MS Method

We have applied a targeted quantitative metabolomics approach to analyze the samples using a combination of direct injection mass spectrometry with a reverse-phase LC-MS/MS custom assay. This custom assay, in combination with an ABSciex 4000 QTrap (Applied Biosystems/MDS Sciex) mass spectrometer, can be used for the targeted identification and quantification of up to 207 different endogenous metabolites including amino acids, acylcarnitines, biogenic amines & derivatives, uremic toxins, glycerophospholipids, sphingolipids and sugars. The method combines the derivatization and extraction of analytes, and the selective mass-spectrometric detection using multiple reaction monitoring (MRM) pairs. Isotope-labeled internal standards and other internal standards are used for metabolite quantification. The custom assay contains a 96 deep-well plate with a filter plate attached with sealing tape, and reagents and solvents used to prepare the plate assay. First 14 wells were used for one blank, three zero samples, seven standards and three quality control samples. For all metabolites except organic acid, samples were thawed on ice and were vortexed and centrifuged at 13,000x *g*. Then, 10 µL of each sample was loaded onto the center of the filter on the upper 96-well plate and dried in a stream of nitrogen. Subsequently, phenyl-isothiocyanate was added for derivatization. After incubation, the filter spots were dried again using an evaporator. Extraction of the metabolites was then achieved by adding 300 µL of extraction solvent. The extracts were obtained by centrifugation into the lower 96-deep well plate, followed by a dilution step with MS running solvent.

For organic acid analysis, 150 µL of ice-cold methanol and 10 µL of isotope-labeled internal standard mixture was added to 50 µL of serum sample for overnight protein precipitation. Then it was centrifuged at 13000x *g* for 20 min and 50 µL of supernatant was loaded into the center of wells of a 96-deep well plate, followed by the addition of 3-nitrophenylhydrazine (NPH) reagent. After incubation for 2h, BHT stabilizer and water were added before LC-MS injection.

Mass spectrometric analysis was performed on an ABSciex 4000 Qtrap tandem mass spectrometry instrument (Applied Biosystems/MDS Analytical Technologies, Foster City, CA) equipped with an Agilent 1260 series UHPLC system (Agilent Technologies, Palo Alto, CA). The samples were delivered to the mass spectrometer by a LC method followed by a direct injection (DI) method. Data analysis was done using Analyst software version 1.6.2.

Bacteriome Analysis

Raw data processing followed the IMNGS pipeline [6] based on the UPARSE approach [7]. Sequences were demultiplexed, trimmed to the first base with a quality score < 3 and then paired. Reads with less than 300 (for skin swab samples: 250) and more than 600 nucleotides and paired reads with an expected error > 3 were excluded from the analysis. Additional trimming of remaining reads was done by removing 5 nucleotides on each end to avoid GC bias and non-random base composition. A table of zOTUs was constructed by considering all reads before any quality filtering. zOTUs (zero-radius OTUs) are valid operational taxonomic units that provide the maximum possible biological resolution [8]. A cut-off of 0.25% (e.g., deleting any zOTU not reaching this level in any sample) was used in order to avoid spurious zOTUs [9]. Taxonomy was assigned at an 80% confidence level using the SILVA ribosomal RNA gene database project [10]. Data analysis was conducted in the R programming environment using the Rhea R-package and NAMCO microbiome explorer [11,12]. To normalize the absolute read counts, the minimum sum counts were used within samples to calculate diversity. Sequencing depth was evaluated via rarefaction curves, and samples of low quality (n=3 rural fecal samples) were excluded from the analysis. Group diversity was assessed using β-diversity based on generalized UniFrac distances or Bray-Curtis dissimilarity, and α-diversity was determined based on species richness and Shannon effective number

of species diversity. P-values were computed using ANOVA on ranks and corrected for multiple comparisons following the Benjamini-Hochberg method. The impact of covariates on differences in the microbial profile across the entire study cohort was assessed through multivariate permutational analysis. This analysis was performed using the R function "adonis" from the vegan package version 2.5-6. The statistical significance was determined with a significance threshold of $P \leq 0.05$. For fecal samples, taxa with a prevalence of at least 30% in a given group were considered for statistical analysis. Differences between groups at genus level were examined using the linear discriminant analysis (LDA) effect size (LEfSe) method [13] (with default settings available at https://huttenhower.sph.harvard.edu/galaxy/root). LEfSe employs a two-tailed non-parametric Kruskal-Wallis test to assess the significance of variations between two groups. Differences in gut microbiota were considered significant, if they exhibited a p-value of < 0.05 and an LDA score (log10) exceeding 3. Correlation analysis between dietary components and fecal metabolites was performed using Pearson correlation method implemented in the Rhea pipeline. The centered log-ratio transformation is used to remove the compositional constraints from the taxonomic variables [14]. In addition, taxonomic zeros (relative abundance of taxonomic variables with the value zero) were treated as missing data and were excluded from the calculation of correlations. Following this transformation of taxonomic variables, the table is centered and scaled, to adjust for differences in the offset and fold changes respectively, and the Pearson correlation for all pairs is calculated. The significance before and after FDR correction following the Benjamini-Hochberg method is reported together with the number of observations that support the correlation. The color of circles indicates the type of correlation (positive/negative) and radius of circles is proportional to the correlation.

Viral DNA Extraction and Sequencing

Fecal samples (rural n=19, urban n=9, due to limited sample numbers and amounts) were mixed with PBS 1:10 w/v, vigorously vortexed for 1h, and centrifuged at 6000xg for 30 minutes to separate VLPs from bacteria and organic biological matter. The supernatants were subsequently passed through 0.22 um syringe filters to remove the remaining bacteria. The filtrate was further concentrated to 500ul by 10 kDa Amicon Ultra Centrifugal Filters (Merck Millipore, Germany). The concentrates were treated with DNase I for 1h at 37°C to remove non-viral DNA. DNase I was inactivated by heating the concentrates at 70° C for 15 minutes. The samples were incubated at −80 °C for 1 h and 55 °C for 5 min to break down the viral capsid. This was followed by adding 10ul of protease K (20 $mg.mL^{-1}$) to complete the lysis. Protease K was then deactivated at 70°C for 15 minutes. The viral DNA was purified using 1.8× sample volume Agencourt AMPure beads (A63880, Beckman Coulter) according to the manufacturer's instructions. Each sample was finally resuspended in 100 µl sterile water. The extracted DNA was purified using the Genomic DNA Clean & Concentrator (D4064, ZYMO research) according to the manufacturer's protocol. The concentration of the purified DNA was measured via Qubit and amplified using REPLI-g Kit (150023, Qiagen) according to the manufacturer's protocol. The amplified viral DNA were sequenced using Illumina Novaseq 6000 at Genomics Core Facility, Helmholtz Centre Munich.

Viral metagenome Assembly and Analysis

Raw reads produced by sequencing were filtered using fastp [15] to remove adaptors and low-quality bases. Dedupe.sh from the bbmap version 38.79 [16] was used to remove duplicate reads. The remaining reads were then assembled using metaSPAdes with k-mer size set to 21,33,55,77,99 [17]. Contigs longer than 3000 bp from all samples were combined into a contig library (cclib). CheckV v0.8.1 [18] was used to remove flanking host region of proviruses and assess the completeness and quality of generated contigs. To remove redundancy in the contig library, we clustered contigs into a non-redundant contig library (nrclib) using the "rapid genome clustering based on pairwise ANI" protocol in CheckV [18]. The longest contig from each cluster sharing more than 95% identity and 80% coverage were selected as the representative in the nrclib. We then mapped the reads to nrclib using minimap2 [19], and the abundance of each contig was calculated using CoverM v0.6.1 (https://github.com/wwood/CoverM). We used VirSorter2 v2.2.3 [20] to identify viral contigs (VCs) in nrclib. Contigs classified as categories 1 and 2 by VirSorter2 were selected for further analyses. DRAM-v [21] was used to annotate the identified VCs. The taxonomy assigned to these contigs using vConTACT2 [22] and a search against NCBI viral RefSeq

database through the MMSeqs2 taxonomy module [23]. IPHoP [24] was used to predict host of the VCs with default parameters, which is an integrated machine-learning framework for maximizing host prediction of viral sequences. iPHoP can reliably predict host taxonomy at the genus level with a low false discovery rate (FDR <10%). VC replication cycle were predicted using BACPHLIP v0.9.6 [25], which is a robust tool commonly used to predict phage replication cycles in metagenomes with shown accuracy of 98%.

For the statistical analyses performed in R v.4.2.1 (R Core Team, 2022), the following packages were used: *Deseq2* [26] package for differential abundant analyses, the *vegan* package (https://github.com/vegandevs/vegan) was used to calculate the Shannon diversity (using the diversity() function), NMDS of Bray-Curtis dissimilarity (using metaMDS() function), and PERMANOVA for determining significant differences in Bray-Curtis dissimilarities (using adonis() function) using default parameters, *corrplot* package (https://github.com/taiyun/corrplot) for correlation analyses, Pearson correlation was used for analyzing the correlation between viral contigs, dietary components, and fecal metabolites, ggplot2 (https://github.com/tidyverse/ggplot2) package was used for creating graphics.

SUPPLEMENTARY FIGURES

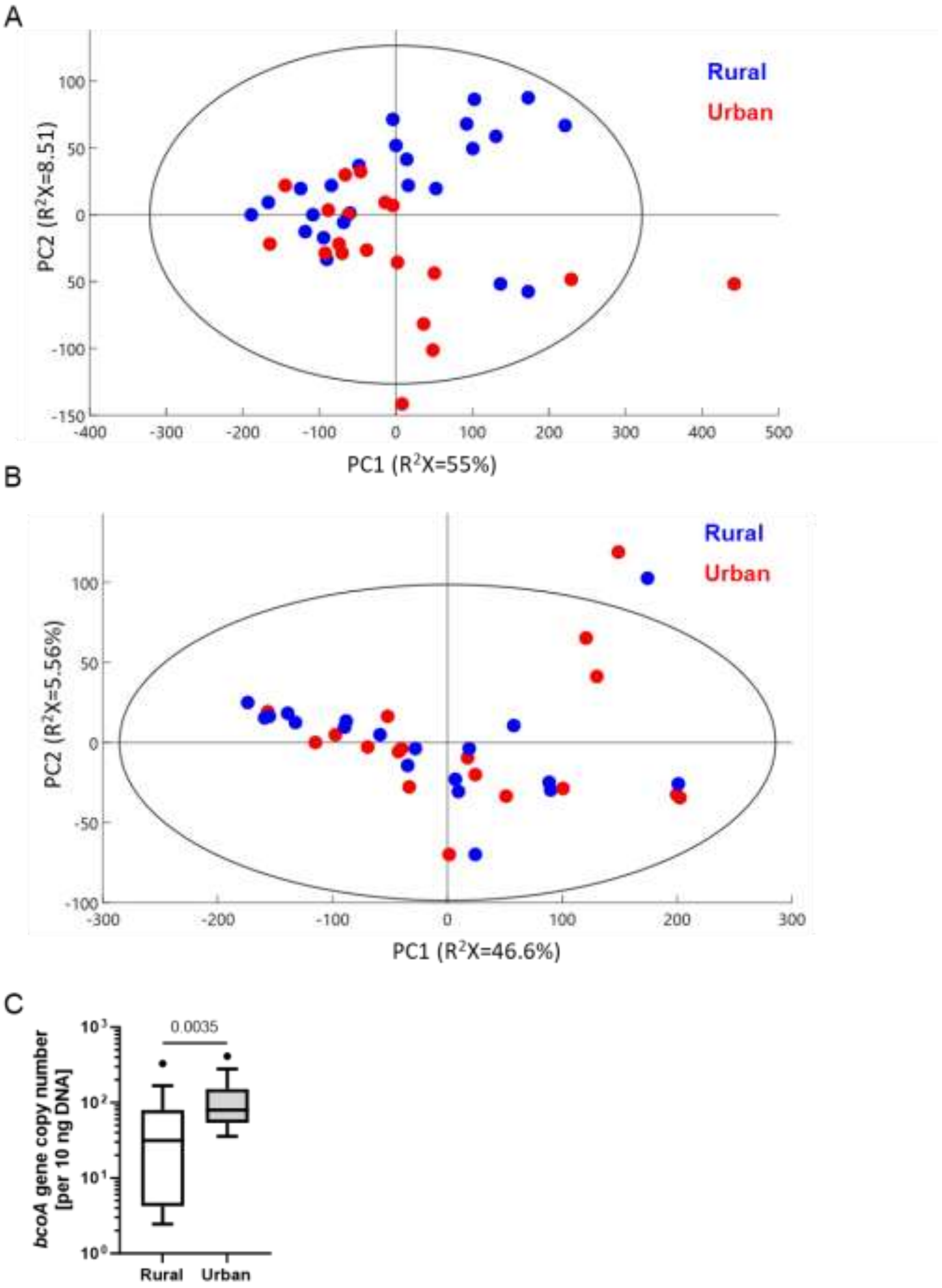

Supplementary Figure 1. Fecal water and urine metabolic profiles show no significant differences between rural and urban Xhosa cohorts

Principal component analysis (PCA) scores plots of ^{1}H NMR spectra of (**A**) fecal water or (**B**) urine obtained from rural (blue) and urban (red) Xhosa individuals with R^2X representing percentages of variance explained by each PC. (**C**) Numbers of *bcoA* gene copies in fecal microbial DNA (rural n=21, urban=20) representing bacteria involved in butyrate production. Statistical analysis was performed by two-sided non-parametric Mann-Whitney-U-test (data not normally distributed). A value of $p<0.05$ was considered to be statistically significant with **=$p<0.01$. Box-and-whisker plot with whiskers minima and maxima according to Tukey, center as median, box from 25th to 75th percentile.

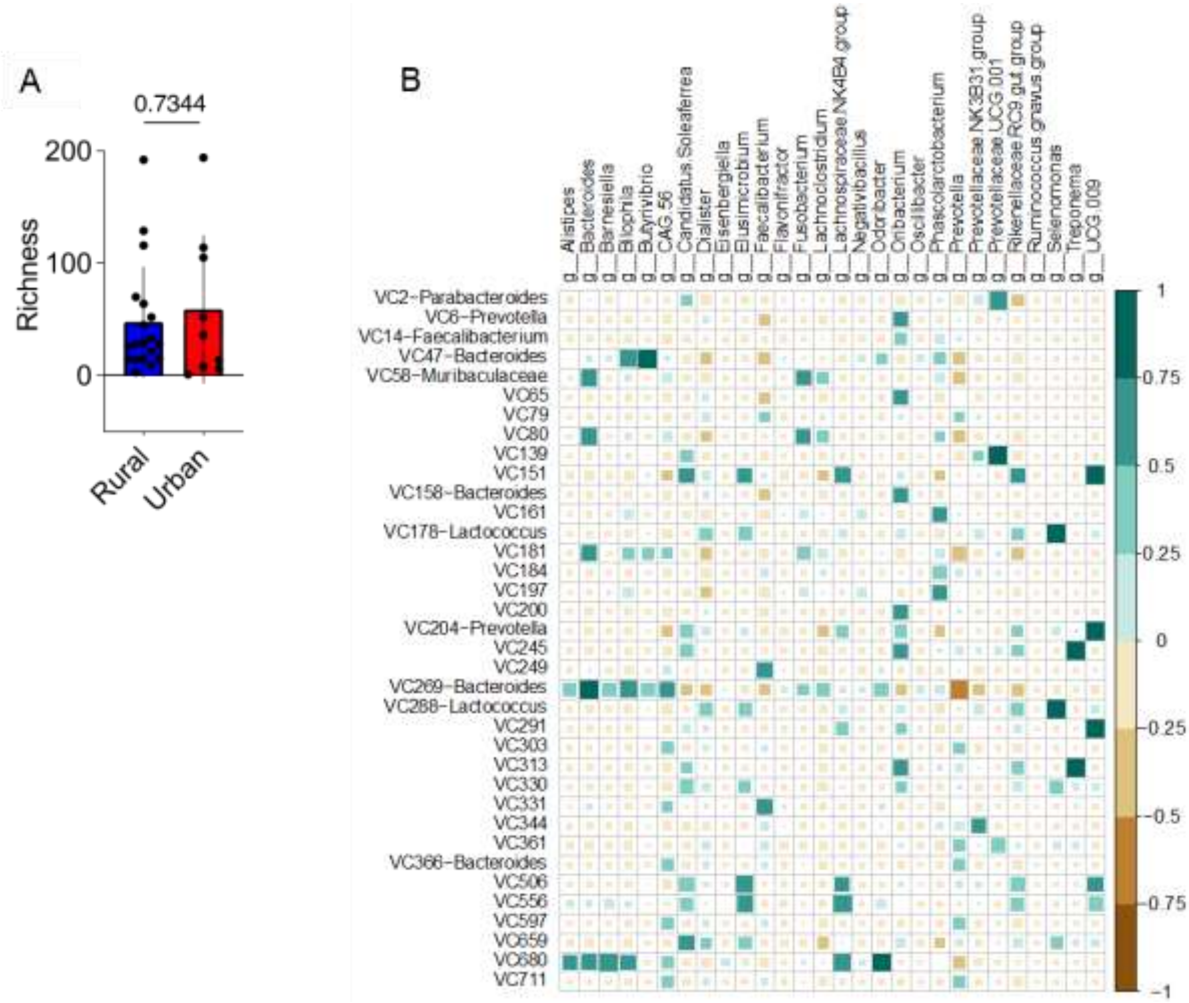

Supplementary Figure 2. No significant differences in viral diversity between rural and urban samples.

(**A**) Differences in VCs evenness and richness between rural (n=19) and urban (n=9) Xhosa fecal samples. (**B**) Pearson's correlation between VCs and bacterial genera differentially abundant in rural or urban Xhosa fecal samples. Statistical analysis was performed by a two-sided Wilcoxon test. A value of *p*<0.05 was considered statistically significant. When present, error bars correspond to the standard deviation of viral richness scores.

SUPPLEMENTARY TABLES

Supplementary Table 1. Concentration of fecal amino acids in rural and urban Xhosa cohorts

**Amino acid**	**Rural**		**Urban**		***P* value**
	**n**	**mean ± SEM**	**n**	**mean ± SEM**	
Glutamine (µmol/g)	24	2.66 ± 0.21	19	2.37 ± 0.23	0.403
Sarcosine (µmol/g)	24	9.97 ± 0.63	19	9.71 ± 0.77	0.875
Glycine (µmol/g)	24	2.14 ± 0.18	19	1.96 ± 0.25	0.389
Alanine (µmol/g)	24	3.46 ± 0.20	19	3.66 ± 0.27	0.690
Serine (µmol/g)	24	1.52 ± 0.17	19	0.84 ± 0.12	0.003**
Valine (µmol/g)	24	3.75 ± 0.29	19	3.27 ± 0.48	0.376
Threonine (µmol/g)	24	1.40 ± 0.13	19	0.97 ± 0.11	0.024*
Phenylalanine (µmol/g)	24	2.46 ± 0.23	19	2.23 ± 0.20	0.620
Taurine (µmol/g)	24	0.11 ± 0.06	19	0.23 ± 0.17	0.469
Ornithine (µmol/g)	24	1.01 ± 0.11	19	0.08 ± 0.09	0.219
Isoleucine (µmol/g)	24	0.83 ± 0.06	19	0.75 ± 0.07	0.389
Glutamic Acid (µmol/g)	24	0.20 ± 0.03	19	0.16 ± 0.02	0.363
Methionine (µmol/g)	24	1.69 ± 0.19	19	1.27 ± 0.16	0.121
Histidine (µmol/g)	23	0.15 ± 0.02	19	0.11 ± 0.01	0.132
Citrulline (µmol/g)	24	0.08 ± 0.01	19	0.08 ± 0.01	0.933
Tyrosine (µmol/g)	24	0.95 ± 0.10	19	0.90 ± 0.11	0.971
Tryptophan (µmol/g)	24	0.16 ± 0.02	19	0.18 ± 0.02	0.490
Cysteine (µmol/g)	8	0.07 ± 0.02	6	0.09 ± 0.06	0.573
Aspartic Acid (µmol/g)	24	0.35 ± 0.05	19	0.35 ± 0.07	0.971
Homocysteine (µmol/g)	12	0.02 ± 0.00	7	0.03 ± 0.01	0.028*
Glutathione (µmol/g)	24	1.10 ± 0.12	19	0.79 ± 0.09	0.045*
Lysine (µmol/g)	24	3.23 ± 0.22	19	2.42 ± 0.20	0.014*
Cystathionine (µmol/g)	21	0.01 ± 0.00	15	0.01 ± 0.00	0.391
Total amino acids (µmol/g)	24	37.24 ± 2.17	19	33.03 ± 2.27	0.185

Values are shown as mean ± SEM (standard error of the mean). Statistical analysis using two-sided unpaired t-test (data normally distributed) or non-parametric Mann-Whitney-U-test (data not normally distributed) with rural n = 24 and urban n = 20 samples. For smaller n-numbers, samples were below the detection limit. Statistical significance highlighted as *=p<0.05, **=p<0.01.

Supplementary Table 2. Concentrations of carboxylic acids in 24h food samples collected from rural and urban Xhosa cohorts[1]

**Metabolite**	**Rural**	**Urban**	***P* value**
Shikimic acid (µmol/g)	0.016 ± 0.003	0.008 ± 0.001	0.038*
Glyeric acid (µmol/g)	0.036 ± 0.012	0.015 ± 0.002	0.138
beta-Hydroxybutyric acid (µmol/g)	0.008 ± 0.007	0.002 ± 0.001	0.608
Lactic acid (µmol/g)	5.680 ± 1.838	1.078 ± 0.782	0.051
Propionic acid (µmol/g)	0.517 ± 0.125	0.417 ± 0.092	0.181
Malic acid (µmol/g)	0.916 ± 0.284	0.746 ± 0.244	0.665
Butyric acid (µmol/g)	0.032 ± 0.004	0.022 ± 0.001	0.047*
Succinic acid (µmol/g)	0.366 ± 0.000	0.094 ± 0.045	0.035*
Fumaric acid (µmol/g)	0.044 ± 0.009	0.014 ± 0.004	0.016*
Valeric acid (µmol/g)	0.003 ± 0.001	0.001 ± 0.000	0.138
Benzioc acid (µmol/g)	0.081 ± 0.030	0.021 ± 0.020	0.051
Oxalic acid (µmol/g)	0.442 ± 0.127	0.765 ± 0.610	0.073
Oxaloacetic acid (µmol/g)	0.270 ± 0.112	0.240 ± 0.058	0.834
Citric acid (µmol/g)	1.051 ± 0.429	0.879 ± 0.243	>0.999
Aconitic acid (µmol/g)	0.010 ± 0.002	0.002 ± 0.001	0.0092**

Pyruvic acid (µmol/g)	0.039 ± 0.027	0.012 ± 0.009	>0.999
alpha-Ketoglutaric acid (µmol/g)	0.025 ± 0.005	0.006 ± 0.002	0.0097**

[1]Values are shown as mean ± SEM (standard error of the mean). Statistical analysis for rural *n* = 6 and urban *n* = 7 samples using two-sided unpaired t-test (data normally distributed) or non-parametric Mann-Whitney-U-test (data not normally distributed) with statistical significance highlighted as *=*p*<0.05, **=*p*<0.01. The following metabolites were measured, but not included in the table because their values in majority of sample were below detection limit: HPHPA, 5-Hydroxyindoleacetic acid, Hippuric acid, Glutaric acid, Methylmalonic acid, Indoleacetic acid, Salicylic acid, Abscisic acid, Jasmonic acid, and *para*-Hydroxyphenylacetic acid.

Supplementary Table 3. Significantly different abundant bacterial genera or families on hand skin swabs from rural or urban Xhosa cohorts

**Genus**	**Rural**	**Urban**	***P* value**
**Higher relative abundance in rural samples**			
Actinomycetospora	0.21036	0.01133	0.0017**
Bacillus	1.31579	0.07027	0.0023**
Lactococcus	1.32312	0.08371	0.0082**
Lysinibacillus	0.25876	0.01208	0.026*
Marmoricola	0.08951	0	0.045*
Methylobacterium-Methylorubrum	0.28968	0.00468	0.015*
Novosphingobium	0.06953	0	0.045*
Ochrobactrum	0.11746	0.00467	0.033*
Peptoniphilus (family)	0.68552	0.00598	0.029*
Peptostreptococcaceae (family)	1.24073	0.08648	0.022*
Serratia	0.14491	0	0.045*
Sphingomonas	0.86835	0.05624	0.0053**
Weeksellaceae (family)	0.12065	0	0.045*
Williamsia	0.08909	0	0.018*
Weissella	0.68552	0.00598	0.0032**
Williamsia	0.08909	0	0.018*
**Higher relative abundance in urban samples**			
Acinetobacter	2.79783	8.77636	0.022*
Aerococcus	0.07531	0.81730	0.026*
Alkanindiges	0	0.12449	0.018*
Aquabacterium	0.01198	0.16967	0.024*
Collinsella	0.00719	0.12577	0.024*
Desemzia	0.0024	0.14696	0.0021**
Flaviflexus	0	0.08439	0.0056**
Finegoldia (family)	0.03951	0.22644	0.015*
Jeotgalicoccus	0.05217	0.25652	0.027*
Paracoccus	0.06637	1.35231	0.012*
Prevotella	0.30681	3.13780	0.022*
Planomicrobium	0.00689	0.34405	0.024*
Psychrobacter	0.02799	2.6579	0.0032**
Ruminococcus	0.00479	0.09698	0.042*
Thermomonas	0.00487	0.29878	0.01*
Trichococcus	0.00244	0.09474	0.0034**
Succinivibrio	0	0.64904	0.018*
Vibrio	0.00201	0.33056	0.042*

Values are shown as mean. Statistical analysis for rural *n* = 6 and urban *n* = 7 samples using Wilcoxon Rank Sum test (two-sided) adjusted according to Benjamini-Hochberg method with statistical significance highlighted as *=*p*<0.05, **=*p*<0.01.

SUPPLEMENTARY REFERENCES

1. Stansert Katzen, L. *et al.* Home visits by community health workers in rural South Africa have a limited, but important impact on maternal and child health in the first two years of life. *BMC Health Services Research* **20**, 594 (2020).
2. Gaunt, C. B. Are we winning? Improving perinatal outcomes at a deeply rural district hospital in South Africa. *S Afr Med J* **100**, 101–104 (2010).
3. O'Keefe, S. J. D. *et al.* Fat, Fibre and Cancer Risk in African Americans and Rural Africans. *Nature Communications* **6**, (2015).
4. Veselkov, K. A. *et al.* Recursive segment-wise peak alignment of biological 1H NMR spectra for improved metabolic biomarker recovery. *Analytical Chemistry* **81**, 56–66 (2009).
5. Dieterle, F., Ross, A., Schlotterbeck, G. & Senn, H. Probabilistic quotient normalization as robust method to account for dilution of complex biological mixtures. Application in1H NMR metabonomics. *Analytical Chemistry* **78**, 4281–4290 (2006).
6. Lagkouvardos, I. *et al.* IMNGS: A comprehensive open resource of processed 16S rRNA microbial profiles for ecology and diversity studies. *Scientific Reports* **6**, 1–9 (2016).
7. Edgar, R. C. UPARSE : highly accurate OTU sequences from microbial amplicon reads. *Nature communications* **10**, (2013).
8. Edgar, R. C. UNOISE2: improved error-correction for Illumina 16S and ITS amplicon sequencing. *bioRxiv* 81257 (2016) doi:10.1101/081257.
9. Reitmeier, S. *et al.* Handling of spurious sequences affects the outcome of high-throughput 16S rRNA gene amplicon profiling. *ISME Communications* **1**, 31 (2021).
10. Yilmaz, P. *et al.* The SILVA and 'all-species Living Tree Project (LTP)' taxonomic frameworks. *Nucleic Acids Research* **42**, 643–648 (2014).
11. Dietrich, A. *et al.* Namco: a microbiome explorer. *Microb Genom* **8**, mgen000852 (2022).
12. Lagkouvardos, I., Fischer, S., Kumar, N. & Clavel, T. Rhea : a transparent and modular R pipeline for microbial profiling based on 16S rRNA gene amplicons. *PeerJ* (2017) doi:10.7717/peerj.2836.
13. Segata, N. *et al.* Metagenomic biomarker discovery and explanation. *Genome biology* **12**, R60–R60 (2011).
14. Aitchison, J. The Statistical Analysis of Compositional Data. *Journal of the Royal Statistical Society. Series B (Methodological)* **44**, 139–177 (1982).
15. Chen, S., Zhou, Y., Chen, Y. & Gu, J. fastp: an ultra-fast all-in-one FASTQ preprocessor. *Bioinformatics* **34**, i884–i890 (2018).
16. Bushnell, B. *BBMap: A Fast, Accurate, Splice-Aware Aligner*. https://www.osti.gov/biblio/1241166 (2014).
17. Bankevich, A. *et al.* SPAdes: A New Genome Assembly Algorithm and Its Applications to Single-Cell Sequencing. *J Comput Biol* **19**, 455–477 (2012).
18. Nayfach, S. *et al.* CheckV assesses the quality and completeness of metagenome-assembled viral genomes. *Nat Biotechnol* **39**, 578–585 (2021).
19. Li, H. Minimap2: pairwise alignment for nucleotide sequences. *Bioinformatics* **34**, 3094–3100 (2018).
20. Guo, J. *et al.* VirSorter2: a multi-classifier, expert-guided approach to detect diverse DNA and RNA viruses. *Microbiome* **9**, 37 (2021).
21. Shaffer, M. *et al.* DRAM for distilling microbial metabolism to automate the curation of microbiome function. *Nucleic Acids Research* **48**, 8883–8900 (2020).
22. Bin Jang, H. *et al.* Taxonomic assignment of uncultivated prokaryotic virus genomes is enabled by gene-sharing networks. *Nat Biotechnol* **37**, 632–639 (2019).
23. Mirdita, M., Steinegger, M., Breitwieser, F., Söding, J. & Levy Karin, E. Fast and sensitive taxonomic assignment to metagenomic contigs. *Bioinformatics* **37**, 3029–3031 (2021).
24. Roux, S. *et al.* *iPHoP: an integrated machine-learning framework to maximize host prediction for metagenome-assembled virus genomes*. http://biorxiv.org/lookup/doi/10.1101/2022.07.28.501908 (2022) doi:10.1101/2022.07.28.501908.
25. Hockenberry, A. J. & Wilke, C. O. BACPHLIP: predicting bacteriophage lifestyle from conserved protein domains. *PeerJ* **9**, e11396 (2021).
26. Love, M. I., Huber, W. & Anders, S. Moderated estimation of fold change and dispersion for RNA-seq data with DESeq2. *Genome Biology* **15**, 550 (2014).
