# Supplementary material for: Diet changes due to urbanization in South Africa are linked to microbiome and metabolome signatures of Westernization and colorectal cancer
Source: Nat Commun. 2024 Apr 20;15:3379. doi: 10.1038/s41467-024-46265-0 (PMC11032404; doi:10.1038/s41467-024-46265-0)
Supplement: Supplementary file 4 — Description of Additional Supplementary Files [file 41467_2024_46265_MOESM4_ESM.pdf]

Description of Additional Supplementary Files

File Name: Supplementary Data 1

Description: Metadata of study participants including dietary assessment, fecal short-chain fatty acids and fecal bile acids.
